# Interferon-induced transmembrane proteins in cancer and virus biology: a membrane perspective

**DOI:** 10.1128/mbio.00784-26

**Published:** 2026-06-17

**Authors:** Tingting Xia, Andrea Cimarelli

**Affiliations:** 1 Centre International de Recherche en Infectiologie (CIRI), Université de Lyon, Inserm, U1111, Université Claude Bernard Lyon 1, CNRS, UMR5308, École Nationale Supérieure de Lyon 133614, Lyon, France; The Ohio State University, Columbus, Ohio, USA

**Keywords:** IFITM, IFITM3, membrane, interferon, PI3K, RTK, STAT, cancer

## Abstract

The interferon-induced transmembrane proteins (IFITMs) are a family of potent broad-spectrum antiviral factors that restrict enveloped viruses primarily by altering the biophysical properties of membranes, thereby preventing viral-cellular fusion. While their antiviral mechanisms are well established, increasing evidence suggests that IFITMs can also play important roles in cancer biology by remodeling of the cancer cell surface proteome and by modulating signaling pathways involved in proliferation, cell survival, and metastasis. In this review, we summarize the diverse biological functions attributed to IFITMs over the years; we highlight the salient aspects of their biology; and we propose a unifying conceptual framework in which seemingly diverse functions of IFITMs in virology and cancer biology can be understood through their capacity to regulate the behavior of membranes.

## INTRODUCTION

## AN OVERVIEW OF THE IFITM FAMILY

**Fig 1:**
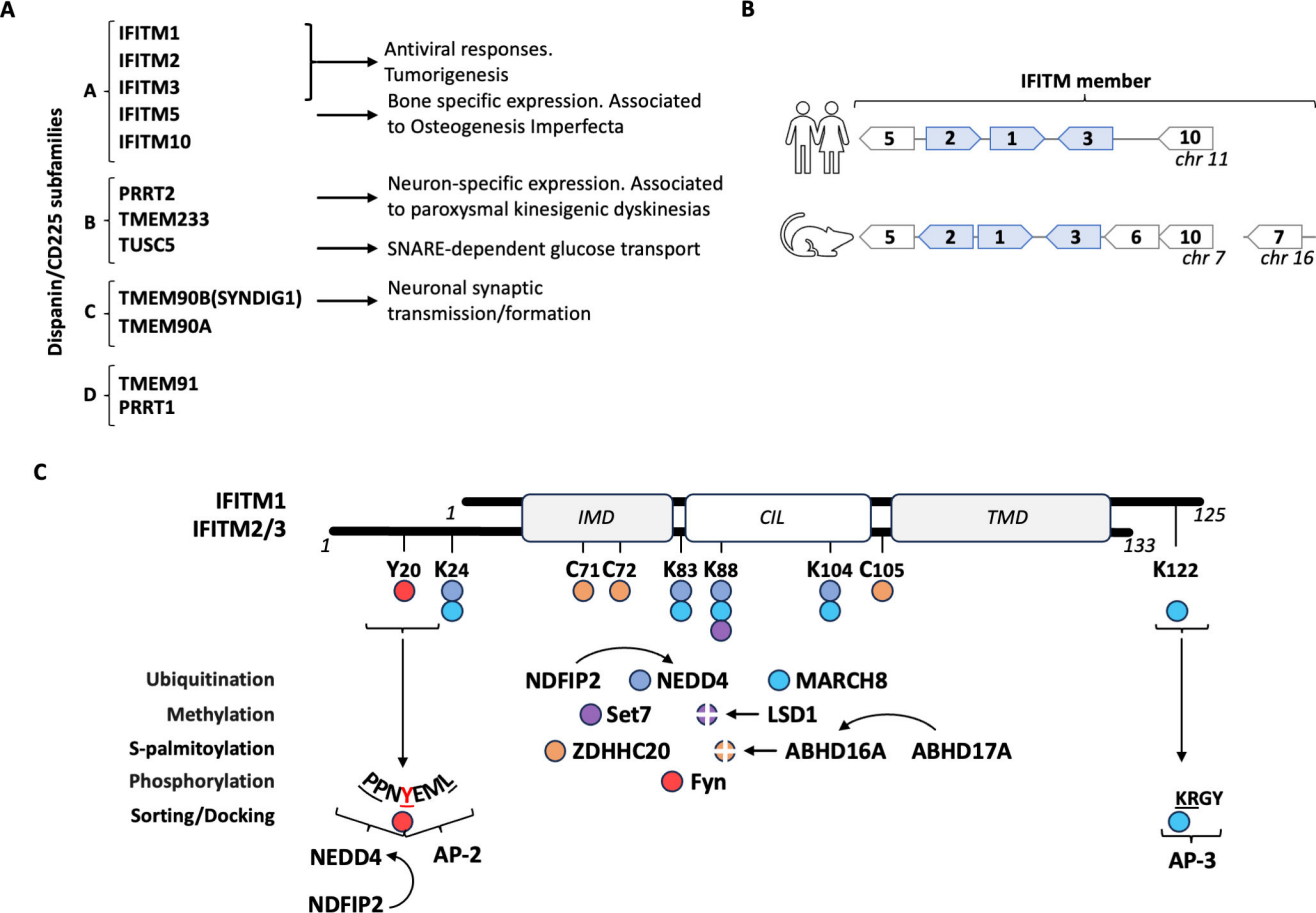
CD225/dispanin family and IFITMs. (**A**) Schematic presentation of the CD225/dispanin protein family and subfamilies. The biological functions associated to known members are highlighted. (**B**) Genomic organization of IFITM genes in the human and mouse genome. Transcriptional .is represented by the direction of the arrow. (**C**) Schematic representation of the IFITM protein topology and of the IFITM-associated proteome. Specific post-translational modifications listed on the left of the figure are color-coded. The position is shown on the specific IFITM amino acid residue (numbering refers to human IFITM3 for which most of the data have been produced, with the exception of K122, which is present only on human IFITM1). Enzymes that promote it or remove it are represented with a full or barred circle, respectively, using the same color code as above. When known, the second line of protein partners validated for a modulatory role of the effects of IFITM proteins is also shown. IMD, intramembrane domain; CIL, cytoplasmic internal loop; TMD, transmembrane domain. Numbers refer to amino acid length of human IFITMs.

The interferon-induced transmembrane proteins (IFITM) are part of the dispanin/CD225 family. Members of this family are defined as proteins bearing two intra- or trans-membrane helices (1) further subdivided into four subfamilies (A through D, Fig. 1A). Among its members are proteins with roles related to endosomal trafficking, such as the IFITM proteins themselves, the trafficking regulator of GLUT4 (TUSC5/TRARG1), the proline-rich transmembrane protein 2 (PRRT2), or the synapse-differentiation inducing one protein (TME90B/Syndig1), as well as proteins with poorly characterized functions. In humans, the IFITM family comprises five genes clustered on chromosome 11, that is, IFITM1, IFITM2, IFITM3, IFITM5, and IFITM10, while in mice (*Mus musculus*), there are six IFITMs clustered in chromosome 7 and an additional member in chromosome 16 (Fig. 1B). This review is devoted to IFITM1, IFITM2, and IFITM3 that are regulated by interferon and that represent the most extensively studied IFITM proteins in both antiviral responses and tumor biology. They will be hereafter referred to as IFITMs.

## AN HISTORICAL OVERVIEW OF IFITM PROTEINS

IFITM proteins were first identified in 1984 as genes whose expression was strongly upregulated by type I interferon (2). Initially designed as 9-27 (IFITM1), 1-8D (IFITM2), and 1-8U (IFITM3) (3), the expression of these proteins was later confirmed as transcriptionally driven by IFN-sensitive response elements (ISRE) and IFN gamma-activated sites (GAS) present in their promoters (4). While IFITMs are strongly induced by interferons, their basal expression is extremely heterogeneous across tissues, cell types, and also pathological condition, suggesting functions that, as we will detail below, go beyond interferon. In humans, IFITM2 and IFITM3 share 91% of identity between themselves and around 80% identity with IFITM1. The most conserved portions of these proteins are a hydrophobic intramembrane domain (IMD, previously referred to as TM1), a cytoplasmic internal loop (CIL), followed by a transmembrane domain (TMD, previously referred to as TM2). Instead, the N- and C-termini are variable and represent the most divergent regions among IFITM family members (5) (Fig. 1C). Consistent with their domain architecture, IFITM proteins localize to multiple cellular membranes.

The first functional studies on IFITMs were carried out in the field of murine immunology using antibodies directed against a cell surface antigen, named Leu13a, which was known to be expressed in cultured cells upon IFNγ and IFNα stimulation (6). Later identified as IFITM1, Leu13a was described to be associated in a functional complex involving CD21/CD19 and TAPA-1 and to play an important function during B cell development (7, 8), an involvement that was very recently extended to IFITM3 (9). A potential role for IFITMs in tumorigenesis was proposed in the early 1990s following associative studies of higher expression of IFITM3 in colitis-associated cancer (10). A few years later, murine IFITM proteins (referred to as murine ifitm-like proteins, mil-1, *fragilis*) were proposed to play a role in germ cell homing (11), though this finding remains controversial (12). Then in 2009, following a genetic screen for viral modulators carried out by the Elledge group, IFITMs came to the limelight of antiviral responses as proteins capable of inhibiting a large spectrum of viruses (13). This prominent role in antiviral responses, more in line with their initial discovery, has bloomed ever since.

## THE BIOLOGY OF IFITM PROTEINS

The biology of IFITMs has been extensively explored in the field of virology, which has demonstrated the tight link existing between functions and subcellular localization of IFITMs. This parameter is regulated by a complex interplay between the N- and C-terminal regions of IFITMs that act as docking platforms for cellular partners and by post-translational modifications dispersed across the proteins’ length. For clarity, these two regulatory layers are described separately (Fig. 1C).

### Protein sorting and docking signals in IFITMs

At steady state, IFITM1 is more enriched at the plasma membrane, whereas IFITM2 and IFITM3 are more concentrated in endo-lysosomal compartments. This distribution must not be regarded neither as static nor exclusive because as a consequence of membrane dynamics, IFITM proteins constantly shuttle between intracellular compartments and are, thus, present simultaneously in multiple locations, albeit at different concentrations. In addition, IFITM proteins can form both homo- and hetero-multimers, and this latter property has been described to also contribute to changes in the intracellular distribution of individual IFITM members (14–16).

Despite these considerations, it remains that IFITM1 exhibits a differential distribution when compared to IFITM2 and IFITM3, which is governed by variations in the N- and C-terminal regions of IFITM proteins that serve as docking platforms for components of the membrane-sorting machinery (Fig. 1C).

In IFITM2 and IFITM3, which feature a longer N-terminal region compared to IFITM1, a conserved amino acid stretch spanning residues 17–23 (PPNYEML; numbering according to human IFITM3) acts as a critical regulator of subcellular trafficking by integrating two canonical motifs: a PPxY motif (specifically PPNY) that binds the WW domains of the E3 ubiquitin ligase neural precursor cell-expressed developmentally downregulated protein 4 (NEDD4) and a YxxΦ sorting motif (specifically YEML) that recruits the μ2 subunit of the adaptor protein-2 (AP-2) complex (17). Interestingly, the tyrosine in position 20 (of human IFITM3) shared by both domains can be phosphorylated by the protein kinase Fyn (18) and likely by additional members of the Src family (see below).

While mutations in the PPNY domain of IFITM3 abolish interaction with NEDD4 and lead to increased levels of IFITM3 (17), suggesting that NEDD4 controls the steady-state levels of IFITM3 via ubiquitination, mutations in the YEML domain of IFITM3 lead to accumulation of IFITM3 at the plasma membrane, phenocopying the effects of μ2 subunit depletion. Given the predominant localization of IFITM3 in endo-lysosomal compartments at equilibrium, these findings suggest that IFITM3 initially reaches the plasma membrane before being internalized and redistributed to intracellular membranes (19).

Because of the fact that its N-terminus is shorter and lacks the docking region described above, IFITM1 cannot recruit neither AP-2 nor NEDD4. However, IFITM1 also seems to be regulated by a non-canonical dibasic sorting signal KRXX (122-125, amino-acid numbering refers to human IFITM1) positioned at its C-terminus through which the cellular adaptor protein complex 3 (AP-3) is functionally recruited (20). Given that the human IFITM1 protein is the only member among mammal IFITM1-like proteins to contain this dibasic sorting signal, this result would suggest a very species-specific adaptation that occurred only in the human genome (20, 21).

### Post-translational modifications of IFITMs

As of today, IFITMs have been shown to be modified by tyrosine phosphorylation, cysteine S-palmitoylation, lysine ubiquitination, and lysine methylation (Fig. 1C).

#### Phosphorylation

The tyrosine residue at position 20 (Y20) within the PPNYEML amino-acid stretch (human IFITM3) is phosphorylated by the Fyn kinase (22). Mutation in this residue leads to an increased, albeit not exclusive, redistribution of IFITM3 at the plasma membrane, interferes with IFITM3 ubiquitination (17), and results in decreased antiviral activity against influenza virus (18). While it remains unclear whether these changes stem directly from alterations in phosphorylation itself or from subtle changes in the recruitment of AP-2 or NEDD4, both of which rely on tyrosine for binding, the positioning of Y20 in a complex docking platform is extremely interesting.

#### Palmitoylation

Palmitoylation is a covalent lipid modification that occurs on cysteine residues. Given that it enhances a protein’s affinity for lipid bilayers, this modification influences the association and localization of a protein on membranes (23). Sequence analysis and biochemical validation identified three conserved palmitoylation sites in IFITM3 (C71, C72, and C105), with corresponding sites in IFITM1 and IFITM2, underscoring the conserved nature of this regulatory mechanism. Palmitoylation of these residues controls the localization and antiviral properties of IFITM3 likely by modulating its binding to cholesterol (23, 24). In humans, palmitoylation is catalyzed by around 20 zinc finger DHHC domain-containing palmitoyltransferases (ZDHHCs). In the case of IFITM3, ZDHHC20 has been described as the primary enzyme responsible for IFITM3 palmitoylation, with contributory roles played by ZDHHC3 and ZDHHC7 (25). While most studies have so far focused on IFITM3, research on IFITM1 may provide additional insights into the complexity of this regulatory mechanism. Indeed, S-palmitoylation is a reversible modification, and the depalmitoylase α/β-hydrolase domain-containing 16A (ABHD16A) has been shown to remove palmitate groups from swine IFITM1 (26), reducing its antiviral functions. Given the conservation of cysteine residues across mammalian IFITMs, it is reasonable to hypothesize that similar regulatory mechanisms operate also in human IFITMs. To add to the complexity of this regulatory network, ABHD16A is itself regulated by the depalmitoylase ABHD17A. Overexpression of ABHD17A leads to a surprising increase in IFITM1 palmitoylation, an effect attributed to the ability of ABHD17A to decrease the intracellular levels of ABHD16A, thus relieving its negative regulation of IFITM1 (27).

#### Ubiquitination

Ubiquitination of IFITM3 has been shown to be mediated by the E3 ubiquitin ligase NEDD4 through the PPxY motif mentioned above and, more recently, by the membrane-associated RING-CH 8 protein (MARCH8) in a PPxY-independent manner (28). Human IFITM proteins contain four conserved lysine residues: K24 (N terminus), K83, K88, and K104 (in the CIL, numbering refers to human IFITM3) that are highly conserved across mammalian IFITMs (29). An additional lysine residue (K122) is present in the C terminus of human IFITM1. Interference with NEDD4 or MARCH8 leads to an increase in the steady-state levels of IFITM3, indicating that IFITM3 ubiquitination is degradative (17, 28). However, mutation of IFITM3 lysine residues leads to a perinuclear accumulation of mutant IFITM3 (29), suggesting that ubiquitination may also participate in the protein’s trafficking. In agreement with this contention, K83 and K88 are respectively part and juxtaposed to a signal sequence in the CIL (SVKSRD, position 81-86 of human IFITM3) that regulates the egress of IFITM3 and of other IFITM members from the cis-Golgi (30). Mutations in this domain phenocopy K83 and K88 lysine mutants and accumulate in this organelle, suggesting that ubiquitination could also regulate egress of IFITMs from the Golgi before it reaches other membranes.

Of note, the dibasic sorting motif (KRXX, residues 122–125) in the C-terminus of IFITM1 contains a lysine residue that could be, at least potentially, ubiquitinated. Given that MARCH8 acts independently of a PPxY motif (28), it is tempting to speculate that MARCH8 may more specifically target IFITM1 and influence its recruitment of the AP-3 sorting complex.

#### Methylation

A single lysine residue (K88) in IFITM has been shown to be methylated. This modification has been shown to be added by histone-lysine N-methyltransferase (SET7) and to be removed by lysine-specific demethylase 1 (LSD1) (31, 32). The consequences of lysine methylation have been extensively studied in the context of their influence on the chromatin-associated functions of histones. However, proteomic studies identified hundreds of non-histone proteins post-translationally modified by methylation on lysine and/or arginine residues (33, 34). Methylation can influence protein stability and/or localization, hence modifying the protein’s behavior (35). In the case of IFITMs, interference with the protein mono-methylation decreases the antiviral activity of IFITM3, but it remains unclear whether this occurs through alteration of its half-life or its trafficking dynamics. In addition, it remains unclear to what extent the competition between methylation and ubiquitination on the same residue is important in regulating the functions of IFITM proteins.

Overall, the panorama of proteins that interact and/or modify IFITMs is extremely rich and, by their nature, likely to fine-tune IFITM functions in response to external cues.

This is the case for palmitoylating and depalmitoylating enzymes that can fine-tune the association between IFITMs and membranes; for members of the Src kinase family that may spatially modulate the ability of IFITM proteins to recruit AP-2 through phosphorylation at sites in which they concentrate, such as focal adhesion sites (18), as well as for NEDD4-mediated ubiquitination, which may regulate IFITM levels in response to a number of cofactors, such as the NEDD4 family interacting protein 2 (NDFIP2), an adaptor that locally competes with IFITM3 for NEDD4 binding in lysosomal vesicles (36). How these layers of regulation intertwine in determining the spatial and dynamic behavior of IFITM proteins remains to be fully appreciated.

## ANTIVIRAL FUNCTIONS OF IFITMs

IFITMs are well recognized for their ability to inhibit a very broad spectrum of viruses spanning from *Orthomyxoviridae* (influenza A virus) (13) to *Poxviridae* (Vaccinia virus, VACV) (37). While much of the evidence for IFITM3-mediated viral inhibition comes from *ex vivo* studies, genetic evidence in mice provides compelling support for its importance in limiting morbidity and mortality *in vivo* (38–40). To strengthen this contention, several genetic variations exist at the *ifitm3* locus in the human population, and two single nucleotide polymorphisms (SNPs rs12252-C and rs34481144) have been associated with increased risks of severe respiratory virus infections (38, 41). The first SNP has been hypothesized to lead to the formation of a truncated form of IFITM3, while the second, located in the 5′ untranslated region of the IFITM3 mRNA, has been hypothesized to alter mRNA stability and/or translation, thus influencing the overall amounts of IFITM3 present in the cell.

**Fig 2:**
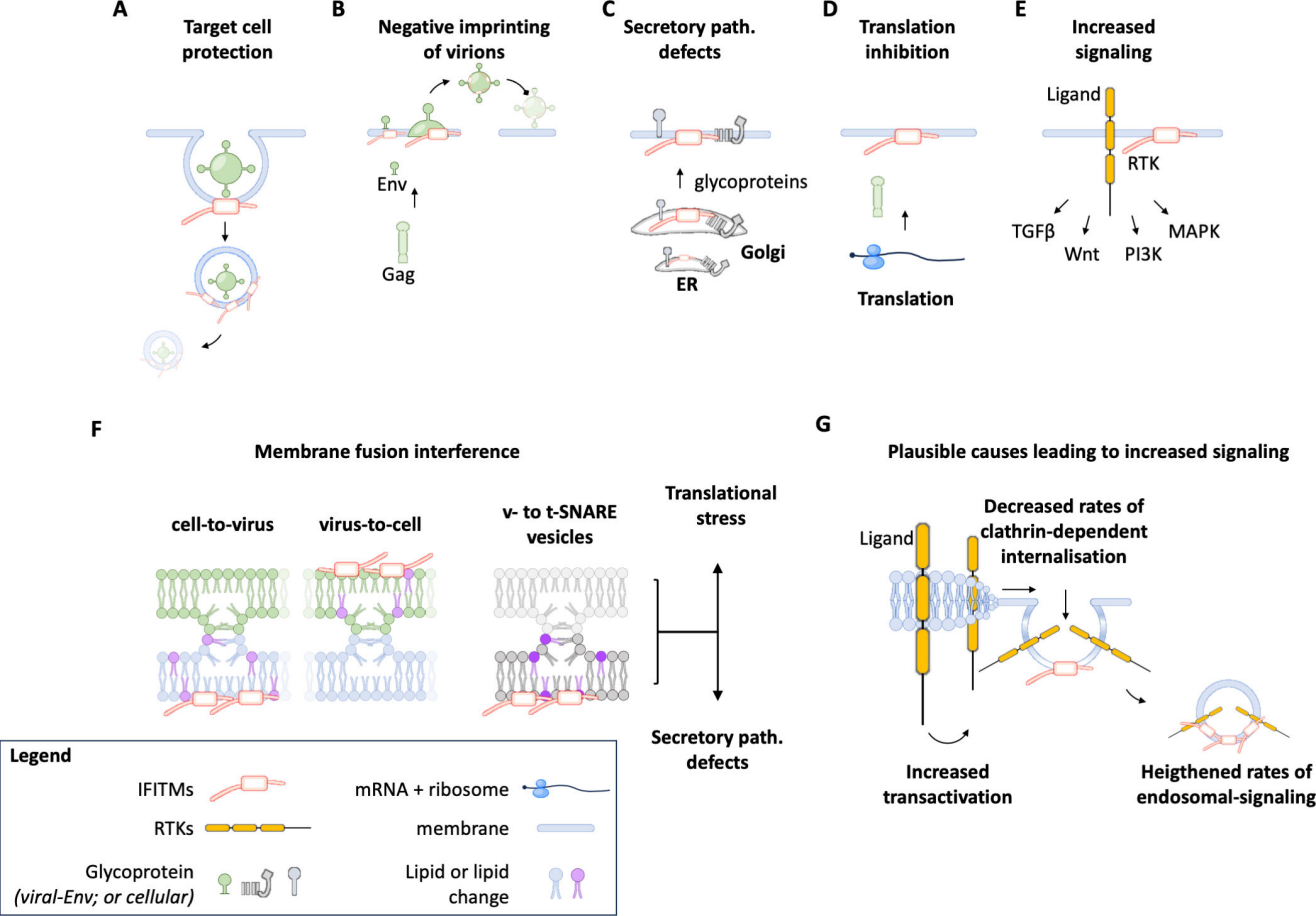
IFITM functions in virus and cancer biology: a membrane-centered explanation. (**A–E**) Functions ascribed to IFITM proteins. More specifically, the mechanisms of inhibition depicted in panels A through D have been described essentially in virus-related studies, while panel E has been essentially observed in tumor-related ones. (**F**) Membrane fusion interference.by IFITMs according to a mixed model of direct insertion and lipid change in the antiviral functions described in panels A, B, or C. Effects of IFITMs on v-to-t-SNARE fusion may lead to global defects described in panels C and D on the cellular secretory pathway and on the translation process. (**G**) Simplified schematic of RTK signaling and endocytosis and possible mechanisms of interference by IFITMs.

The most studied mechanism of viral inhibition by IFITMs is their ability to inhibit the process of viral-to-cell membrane fusion at two distinct steps of the virus life cycle: during viral entry (a property referred to as target cell protection) and during the production of infectious virion particles (negative imprinting of virion particles infectivity). Two additional mechanisms of viral inhibition by IFITMs have also been reported in the interference with viral envelope glycoprotein (Env) processing and in the interference with the process of protein translation (Fig. 2).

### Target cell protection

The first antiviral activity attributed to IFITM proteins was their ability to protect target cells from incoming virion particles by inhibiting the fusion between the viral and the cellular membranes (13), a critical step in the life cycle of all enveloped viruses. Virus entry into the cell can occur through either pH-dependent or -independent pathways. In the pH-dependent route, virions are internalized into acidic compartments, where low pH triggers conformational changes in viral glycoproteins that drive membrane fusion. In contrast, in the pH-independent entry pathway, viruses do not require acidification to undergo fusion and may fuse directly at the plasma membrane.

Endosomally localized IFITMs, and more specifically IFITM2 and IFITM3, which are enriched in these compartments, intercept virion particles entering via endosomes and inhibit the progression of membrane fusion from the hemifusion state to fusion pore formation (42). As a consequence, virions remain trapped within endosomal compartments and are ultimately degraded following endosome–lysosome fusion (Fig. 2A). Elegant kinetic studies have suggested that IFITMs do not impose an absolute block to fusion, but instead delay the process, thereby increasing the likelihood that virion-containing endosomes will fuse with lysosomes before productive fusion can occur (43).

Despite the marked sensitivity of many pH-dependent viruses to IFITM restriction, non-pH-dependent viruses are also susceptible to IFITMs, as for example HIV-1 (44–46). Although the HIV-1 envelope glycoprotein complex (gp120–gp41) drives membrane fusion at the plasma membrane, HIV-1 entry is nonetheless negatively affected by IFITM proteins, including members enriched in endosomes, such as IFITM2 and IFITM3 (47–49). This finding is not overall surprising in light of the fact that HIV-1 particles have also been shown to functionally enter cells via endosomes (50), although this finding remains debated (51), and that membrane dynamics are likely to constantly shuffle IFITM3 between endosomes and plasma membrane.

### Negative imprinting of virion particle infectivity

In 2014 and 2015, our laboratory, together with the Schwartz and Liu laboratories, reported that IFITMs could inhibit an additional step of the viral life cycle of HIV-1 in that IFITM expression in HIV-1-producing cells resulted in progeny virions that incorporated IFITM proteins and exhibited reduced infectivity (44, 46, 52). We termed this phenomenon, which we later described also for a broad range of viruses (53), negative imprinting (Fig. 2B), to reflect a process in which the antiviral effect is “written” into virion particles during assembly but becomes apparent only during the subsequent round of infection when IFITM-virions fail to efficiently fuse with target cells. Several studies did not identify major changes in the protein composition of these virions, except for the incorporation of IFITMs themselves, which are packaged during the process of virion particle formation and egress from the cell (44, 46, 54). However, no direct correlation could be observed between the amount of IFITM incorporated into virion particles and the magnitude of the antiviral effect (47, 55). This suggests that IFITM incorporation into virions is more likely a consequence of their presence in cellular membranes at sites of viral assembly rather than the direct cause of their decreased infectivity. As we will discuss below, this observation bears implications for the mechanism through which IFITMs alter membrane fusion.

### Influence of IFITM proteins on viral envelope processing and trafficking

An additional mechanism through which IFITMs have been described to inhibit viral replication is through interference with viral glycoprotein processing. Viral glycoproteins, just as cellular ones, transit through the ER-Golgi secretory pathway. IFITMs appear to divert part of Env proteins from the canonical ER–Golgi–plasma membrane pathway toward endolysosomal compartments (52, 56) (Fig. 2C), reducing the steady-state abundance of Envelope proteins at viral assembly sites. These defects are in line with a role of IFITM molecules as modulators of the cellular secretory pathway that we have described outside a viral infection context (30).

Changes in the amount of envelope incorporated on virion particles are, however, unlikely to explain the infectivity defect of virions produced in the presence of IFITMs first because such a decrease has not been consistently reported (44, 46, 54), and second because, at least in the case of HIV-1, in which this has been more deeply focused, the efficiency with which a virus enters the cell is not directly proportional to the levels of Env at the surface of the virus particle (57).

### Influence of IFITM proteins on viral translation

IFITM1, IFITM2, and IFITM3 have been reported to reduce HIV-1 Gag expression in infected cells, with the inhibitory effect occurring primarily at the level of viral RNA translation, rather than transcription or RNA stability (58) (Fig. 2D). Interestingly, the HIV-1 non-structural viral protein Nef appears to counteract this inhibition without significantly altering IFITM protein levels. To date, this mechanism of inhibition has not been documented in other viral systems, but it is, however, of interest to note that a proteomic analysis of exosomal vesicles produced in the presence or absence of IFITM3 revealed a relative enrichment of proteins involved in translation in IFITM3-vesicles when compared to control ones (36). Whether this may relate to a functional impairment of translation in cells expressing IFITM3 remains to be determined.

## IFITMs AND CANCER

Initially identified only through associative studies, IFITMs are now recognized as active participants in tumorigenesis across a broad spectrum of human cancers, in the respiratory track (lung adenocarcinoma (59), SCLC (60), and NSCLC (61)), the digestive and urinary systems (gastric [62–64], colorectal [65–67], prostate [68, 69], pancreatic [70, 71], and hepatocellular carcinomas [72, 73]), or renal clear cell carcinoma (74), in addition to breast cancer (75), glioma (76, 77), osteosarcoma (78), and myeloid leukemia (79, 80). In the majority of cancer types, albeit not all, IFITM overexpression is functionally linked to tumorigenesis (62, 79, 81). However, the link between cancer and IFITM expression appears to be context-dependent (69, 82, 83), and for example, overexpression of IFITM1 in the context of interferon gamma stimulation has been shown to be responsible for cell growth arrest (84). Overall, and with few exceptions, the molecular mechanism(s) through which IFITM proteins influence the process of tumorigenesis remains descriptive when compared to the field of virology.

### Modification of cell signaling by IFITMs in tumoral cells

A common feature associated with IFITM overexpression in cancer cells seems to lie in their ability to sustain mitogenic signaling, promote cell cycle dysregulations, and protect cells from apoptosis (Fig. 2E). In breast cancer, for instance, IFITM1 overexpression enhances estrogen-independent growth and tumor aggressiveness, and inversely, its silencing markedly reduces both cell proliferation and cell migration (85, 86). Similarly, IFITM2 expression is associated with higher IGF1/STAT3, TGF-β/SMAD2/3, PI3K/AKT, p38/MAPK, or Wnt/β-catenin signaling pathway activation (64), and IFITM3 increases the CCND1–CDK4/CDK6–pRB signaling axis in oral squamous cell carcinoma (OSCC) (59, 87). These pro-oncogenic effects are closely linked to cell cycle dysregulation and can be reversed upon IFITM1 and IFITM3 silencing (63, 71, 73, 77).

IFITM expression also plays a role in epithelial–mesenchymal transition (EMT), a critical process in metastatic progression (61, 88). IFITMs sustain TGF-β1-driven EMT by enhancing SMAD2/3 phosphorylation (68, 89) and lead to MMP2/MMP9 expression across multiple tumor contexts, likely facilitating matrix degradation and cell invasion (73). In agreement with this contention, loss of IFITM1 and/or IFITM3 significantly impairs migration, invasion, and sphere formation in cancer cells (71, 77, 85, 86).

While in most studies, the molecular mechanisms through which IFITM proteins promote signaling remain unclear, an elegant study described how IFITM3 promotes pro-tumoral signaling in B cell malignancies (9). Phosphorylation of tyrosine 20 triggers the relocalization of IFITM3 from endosomes to the plasma membrane, where it binds and concentrates the phospholipid PIP3 via its lysine residues, resulting in enhanced PI3K signaling. This model is of interest for two reasons. First, it proposes that tyrosine phosphorylation acts as a molecular switch redirecting IFITM3 from its canonical antiviral role toward pro-tumoral functions. Second, by linking PIP3 scaffolding to both Src family kinase-mediated phosphorylation and lysine residues susceptible to ubiquitination, it raises the broader and largely unaddressed question of how the interplay between multiple post-translational modifications collectively shapes the cellular functions and dysfunctions of IFITM3.

### Modification of the cell surface proteome by IFITMs in tumoral cells

For the moment, less explored than its signaling effects, IFITM expression has also been associated with altered abundance of certain cell surface proteins, a property that is very interesting in terms of cell behavior, cell adhesion, and invasive properties of tumoral cells. More specifically, IFITM1 has been shown to lead to changes in cell adhesion proteins, such as ALCAM (CD166), MCAM (CD146), and integrins, which may more directly alter the invasive properties of tumors (90). Given that some of these changes are not transcriptional, this suggests that IFITMs may interfere with translation and/or with the functionality of the secretory pathway, influencing the transit of cellular glycoproteins to the cell surface.

## IFITMs AS MODULATORS OF THE BEHAVIOR OF MEMBRANES

The major antiviral function of IFITM proteins lies in their ability to alter the behavior of membranes, inhibiting viral-to-cellular membrane fusion. Although a systematic analysis of all the effects that IFITM proteins can have under endogenous levels of expression is lacking, and despite the fact that these levels are highly cell type dependent, biophysical studies have conclusively demonstrated that membranes of IFITM-expressing cells are more ordered and mechanically rigid, a property that reduces their propensity to undergo fusion. This phenomenon has been demonstrated both in cells and in reconstituted membrane systems using Laurdan-based imaging and lipid-tension reporters, which agree on increased lipid order and stiffness in the presence of IFITM (15, 91–93). Further support for a causal link between altered membrane state and antiviral restriction comes from the observation that amphotericin B, a compound that binds sterols in lipid bilayers and is associated with increased membrane fluidity, relieves the IFITM3-driven membrane fusion defect (94).

At present, two non-mutually exclusive models have been proposed: a direct effect of IFITMs on the membranes in which they are inserted or an indirect effect of IFITMs on the protein and more likely lipid composition of membranes. In our opinion, the co-existence of both models is necessary to explain data in the literature that would be otherwise incoherent (for simplicity, a single membrane presenting both inserted IFITMs and lipid changes is depicted in Fig. 2F).

### Direct effect of IFITMs on membrane behavior

According to this mode of action, IFITMs alter the biophysical behavior of the membranes they insert into, in a manner that is, or rather should be expected to be, directly proportional to their levels of expression in the cell. A direct membrane-remodeling role for IFITMs is supported by the observation that purified IFITM3 rigidifies reconstituted membranes (95, 96), that the antiviral functions of IFITM3 require an amphipathic helix (residues 59–68), a structure known to alter directly the properties of membranes (95, 97, 98), and that IFITMs multimerize (15).

In our opinion, this model does not explain the full extent of the effects of IFITMs that have been reported in the literature. In virion particles incorporating IFITMs, there is a lack of correlation between the levels of IFITM incorporation and the magnitude of the antiviral effects (47, 55, 99). If membrane rigidification resulted solely from local IFITM insertion, one would expect a direct relationship between the amount of IFITM incorporated into virion particles and antiviral potency, which is not observed.

Second, Laurdan measurements have revealed an identical increase in membrane order at the plasma membrane in the presence of IFITM1, IFITM2, or IFITM3 (92). Even in this case, these results cannot be explained solely by a purely stoichiometric mode of action of IFITM into membranes because IFITM2 and IFITM3 are far less abundant at the plasma membrane than IFITM1. Therefore, one would have expected negligible effects on plasma membrane order for these paralogs.

### Indirect effect of IFITMs on membrane behavior

A second mechanism through which IFITMs could indirectly alter the behavior of membranes is by modifying their lipid composition. While a comprehensive lipidomic analysis of IFITM-bearing membranes is lacking, IFITM3 has been shown to interact with vesicle-associated membrane protein-associated protein A (VAPA), an ER-resident adaptor protein that recruits several lipid transfer proteins for lipid transport between the ER and other organelles (100).

The VAPA-IFITM3 interaction has been shown to alter the association between VAPA and the oxysterol binding protein (OSBP), which is the protein that transports cholesterol from the ER to the Golgi, leading to an increased accumulation of cholesterol in membranes (101). This finding is of interest given the well-established role of cholesterol in viral infectivity (102) and the equally well-documented capacity of elevated intracellular cholesterol levels to rigidify membranes (103). In this context, the increased levels of cholesterol would provide a simple explanation for the membrane rigidification behavior associated with IFITM restriction. However, while additional studies have confirmed cholesterol accumulation in the presence of IFITMs (104–107), some have failed to reproduce this finding or have explicitly excluded cholesterol redistribution as the primary mechanism of viral inhibition by IFITM3 (55, 104, 105, 107). As such, the functional contribution of increased cholesterol levels to the antiviral activity of IFITM proteins remains unresolved.

In addition to cholesterol, VAPA is a central hub for the transport of several lipids and is also a very important membrane tether linking the ER to several organelles, including endosomes, lysosomes, the Golgi apparatus, mitochondria, and the plasma membrane. For its role in lipid transport, VAPA acts as a membrane anchor for several otherwise cytoplasmic lipid transport molecules: OSBP, involved in cholesterol transport; ceramide transfer protein (CerT), involved in ceramide transport; and PI/PC transfer protein membrane associated 1 (PITPNM1 or Nir2), involved in the transport of phosphatidylinositol and phosphatidylcholine. As a membrane tether, VAPA interacts with proteins (ACBD4, or STARD3, STARD3NL, or SNX2) present in several vesicles (peroxisomes or endolysosomes, respectively), therefore binding these vesicles in close proximity to the ER in zones in which lipid transfer from one membrane to the other occurs efficiently (extensively reviewed in reference 108). Interference with any of these functions is expected to produce compartment-specific alterations in the lipid composition of membranes, which, in turn, may affect membrane order, trafficking dynamics, and ultimately propensity to undergo membrane fusion. Of interest, recent studies also suggest a connection between IFITMs and phospholipid metabolism, although the causality of these changes remains to be determined (9, 109).

On the whole, we believe that the most parsimonious model of IFITM action should integrate both their direct insertion into membranes and their indirect remodeling of membrane composition. This dual mode of action could expand the action of IFITMs beyond the membranes in which they insert, potentially influencing whole-cell responses to viral infection.

## TRANSPOSING A MEMBRANE-CENTERED EXPLANATION OF THE ANTIVIRAL EFFECTS OF IFITM PROTEINS TO CANCER BIOLOGY

Whether through direct insertion into membranes, lipid modifications, or both, studies in the field of virology have determined that IFITMs are membrane-rigidifying proteins. During antiviral responses, IFITM expression is transiently induced by interferons, peaking during active infection and subsiding upon viral clearance. This relatively narrow window of expression is important because it is becoming increasingly appreciated how the prolonged expression of components of the interferon system is often deleterious for the organism and associated with several pathological conditions, such as interferonopathies, diabetes, and cancer, in which high expression of several interferon-regulated genes appears a recurrent observed feature (110, 111). We believe that persistent expression of a family of proteins that rigidify membranes, such as IFITMs, is likely to exert pervasive effects on the biology of the cell, contributing to tumorigenesis.

As discussed above, the molecular mechanisms underlying the roles of IFITM proteins in tumorigenesis remain largely unresolved. In the paragraphs below, we draw on evidence from the virology literature to propose possible parallels in cancer biology. These connections are necessarily speculative but may help frame novel hypotheses and open new avenues of investigation.

We have previously shown that prolonged expression of IFITM3 leads to its progressive accumulation in the Golgi, driving impaired trafficking of model glycoproteins to the cell surface (30). Transport of glycoproteins through the Golgi occurs through a series of membrane fusion events between vesicles bearing vesicular and target soluble N-ethylmaleimide-sensitive-factor attachment protein receptors (respectively v-SNAREs and t-SNAREs) that collectively promote forward movement of glycoproteins to the plasma membrane (112). IFITMs are well posited to inhibit the transport of glycoproteins through the Golgi, as this process relies basically on membrane fusion (Fig. 2F). Two lines of evidence support this contention: first, incubation of cells with Amphotericin B relieves the glycoprotein transport defect observed in the presence of IFITMs (30); and second, overexpression of v-SNAREs, which is well known to bypass membrane fusion defects at the Golgi (113, 114), also relieves the negative effects of IFITM3 on glycoprotein transport (30). While complete inhibition of the ER-Golgi transport would not be a viable option, more subtle changes in the functionality of the ER-Golgi secretory pathway bear the potential to influence both the quantity and the quality of proteins present at the cell surface, such as receptors, integrins, or metalloproteases, all of which can influence the invasive properties of tumor cells. Furthermore, these changes can alter the relationship with immune cells through acquisition or loss of proteins that modulate directly their recognition by immune cells, or else can influence the tumor microenvironment. Indications that this is the case are present in the literature, and, for instance, IFITM3 expression has been linked to increased MHC-I expression at the cell surface of tumor cells, leading to higher CD8+T cell infiltrates (115). We believe that the effects of IFITMs on protein translation could result from a translational stress consequent to alterations of the secretory pathway, as, for example, an ER-stress response or an unfolded protein response (Fig. 2F). Future studies would need to determine whether this is the case.

The second commonly observed feature associated with IFITM expression in cancer biology is a positive effect on receptor tyrosine kinase (RTK)-driven signaling. RTKs are transmembrane proteins that become activated through dimerization upon interaction with their ligand, driving a cascade of phosphorylation events that trigger several signal transduction pathways with broad effects on the cell physiology (MAPK, PI3K, JAK-STAT, etc.). Following ligand engagement, RTKs are then internalized via clathrin-dependent endocytosis, and in some cases, clathrin-independent one, leading to their degradation or recycling, overall limiting the activation cascade (reviewed in references 116, 117).

As membrane modulators, we hypothesize that IFITMs can influence RTK signaling at one or more of the following steps (Fig. 2G). RTKs are activated by ligands, but ligand-bound receptors can also activate adjacent unliganded receptors in a process referred to as transphosphorylation, which has been well documented for the epidermal growth factor receptor (EGFR) (118, 119). This process can be influenced by several parameters, among which is receptor density. By rigidifying membranes, IFITMs could increase the local density of particular RTKs by increasing the energy required for the lateral movement and dispersion of membrane-embedded receptors and, thus, lead to an increase in the overall efficiency of transphosphorylation.

In addition, IFITMs could influence RTKs activity through decreased rates of clathrin-dependent internalization. This would seem paradoxical for an antiviral factor that welcomes viruses in endosomes, unless a more specific tumoral environment is evoked. IFITM internalization requires the recruitment of the clathrin adaptors AP-2 (or AP-3 in IFITM1), similar to RTKs, and this is regulated by the phosphorylation status of its tyrosine in position 20 by members of the Src family. An IFITM3 mutant that mimics this phosphorylation (Y20E) exhibits an increased distribution at the plasma membrane likely due to a suboptimal recruitment of the AP-2 adaptor (9). Accrued accumulation of IFITMs at the plasma membrane may, thus, rigidify it and decrease the extent of clathrin-dependent endocytosis of RTK, leading to persistent, or higher, RTK signaling.

Finally, although RTK internalization in endosomes is generally viewed as a limiting event, accumulating evidence indicates that RTK signaling is not restricted to the plasma membrane. Internalized EGFRs are enzymatically active, hyperphosphorylated, and associated with Ras-GAP, Shc, Grb2, and mSOS, indicating that they are actively signaling from endosomes (reviewed in reference 120). The specific composition of IFITM-bearing endosomes, which is unknown for the moment, along with the fact that accumulating evidence indicates that RTK signaling is not restricted to the plasma membrane, suggest that IFITMs could lead to increased RTK signaling from endosomal compartments.

In conclusion, while the antiviral activity of IFITM proteins is largely attributed to their membrane-rigidifying properties, the precise mechanism(s) through which IFITMs regulate tumorigenesis remain incomplete. As membrane modulators, we believe IFITMs can participate in tumor progression in numerous manners. In this review, we have put forward two possible connections between IFITMs’ functions in virology and cancer biology: the modulation of the functionalities of the ER-Golgi pathway, which can alter the cell surface proteome, reshaping the cell’s metastatic potential, and the modulation of RTK signaling, which can sustain this potential transcriptionally. Both of these hypotheses involve the known behavior of IFITMs on membranes, and, interestingly, they can be experimentally tested, providing a framework for future investigation.

The dual nature of innate immune factors that can, on one hand, protect the cell from pathogens and, on the other hand, drive cellular dysfunctions, is a recurring theme in biology and represents a unique opportunity in which lessons from one field can, and should, be transposed to the other.
